# Trajectories of Outpatient Service Utilisation of Hypertensive Patients in Tertiary Hospitals in China

**DOI:** 10.3390/ijerph17030852

**Published:** 2020-01-29

**Authors:** Zijing Pan, Wanchun Xu, Zhong Li, Chengzhong Xu, Fangfang Lu, Pei Zhang, Liang Zhang, Ting Ye

**Affiliations:** 1 School of Medicine and Health Management, Huazhong University of Science and Technology, Wuhan 430030, China; peterpanzijing@gmail.com (Z.P.); wanchunxu6@gmail.com (W.X.); lizhong@hust.edu.cn (Z.L.); zhangliang@mails.tjmu.edu.cn (L.Z.); 2 Research Centre for Rural Health Service, Key Research Institute of Humanities & Social Sciences of Hubei Provincial Department of Education, Wuhan 430030, China; 3 Yichang Centre for Disease Control and Prevention, Yichang 443000, China; function68@hotmail.com (C.X.); fangfanglu91@126.com (F.L.); m15827213395@163.com (P.Z.)

**Keywords:** hypertension, latent class growth analysis, medical expenditures, outpatient utilisation pattern

## Abstract

This study aims to identify the characteristics and trajectories of outpatient service utilisation for hypertensive patients in tertiary hospitals. This study also attempts to investigate the determinants of the trajectories of outpatient service utilisation. A total of 9822 patients with hypertension and hypertension-related medical utilisation were recruited in Yichang, China from January 1 to December 31 in 2016. The latent trajectories of outpatient service utilisation were identified through latent class growth analysis. Differences in the demographic characteristics and medical utilisation among patients in different trajectories were tested by one-way ANOVA and chi-square analysis. The predictors of the trajectory groups of outpatient service utilisation were identified through multinomial logistic regression. Four trajectory groups were determined as stable-low (34.7%), low-fluctuating (13.4%), high-fluctuating (22.5%), and stable-high (29.4%). Significant differences were observed in all demographic characteristics (*p* < 0.001) and medical service utilisation variables (*p* < 0.001) among the four trajectories except for inpatient cost (*p* = 0.072). Determinants for outpatient service utilisation patterns include the place of residence, education level, outpatient visit times, inpatient service utilisation, and outpatient cost. Overall, hypertensive patients visiting outpatient units in the tertiary hospital were middle-aged, elderly, and well-educated, and they received poor follow-up services. The four identified latent trajectories have different characteristics and medical utilisation patterns. Trajectory group-based measurements are necessary for hypertension management and economic burden reduction.

## 1. Introduction

Hypertension is recognised as a serious, worldwide public health concern and a great public health challenge because of its high prevalence [1] (40% worldwide in 2010). The number of hypertensive patients is expected to grow to 1.56 billion in 2025 [2,3,4]. The prevalence of hypertension in the U.S. increased slightly from 28.4% in 1999–2000 to 29.3% in 2013–2014 [5,6]. Meanwhile, the overall prevalence of hypertension in Germany increased from 30% in 1998 to 32% in 2008–2011 [7]. The prevalence of hypertension is also extremely high in developing countries, especially in China [8,9]. The prevalence of hypertension among adults in China increased from 18.8% in 2002 [10]. However, another national survey conducted from 2012 to 2014 reported that the prevalence had risen to 23.2% [11]. Furthermore, the prevalence of hypertension is predicted to rise rapidly due to urbanisation and ageing [12,13].

Hypertension is also one of the leading causes of the economic burden of disease [14,15]. The annual direct and indirect costs of hypertension were $47.3 and $3.9 billion (2012–2013), respectively [16]. Hypertension-attributable costs were predicted to rise from $13.9 billion in 2010 to $20.5 billion by 2020 in Canada [17]. The direct cost associated with hypertension in five European countries was predicted to be €51.3 billion over the 10-year time [18]. Moreover, the annual cost of care and the cost of an acute episode caused by hypertension exceed the total health expenditure per capita in low- and middle-income countries, especially China [19]. As reported, the annual direct and indirect costs of hypertension were 11.1 and 10.5 billion in 2016 in mainland China [20].

In response to these challenges, the Chinese government launched the ‘National Public Health Service Programme’ in 2009. One of the major components of this programme is the hypertension management programme with the combination of primary, secondary, and tertiary prevention strategies, including hypertension detection, measurement, treatment, and follow-up [21,22,23]. The hypertension management programme aims to manage primary hypertensive patients who are 35 years old and above through primary healthcare institutions, such as community health centres in urban areas as well as township health centres and village clinics in rural areas. Patients were classified into different groups according to their blood pressure levels, risk factors, and co-morbid conditions for hypertension management [21]. Standardised health management at the primary level for hypertensive patients is a means to control hypertension and is also an approach for encouraging hypertensive patients to utilise health services at primary healthcare institutions.

In China, a large number of hypertensive patients visit the outpatient units of tertiary hospitals because of the defectiveness of the dual referral system and the weak restriction of medical insurance policies. Moreover, regular follow-up visiting occupy most outpatient services for hypertensive patients in tertiary hospitals instead of patients with severe and complicated conditions, hindering the severe and complicated patients from being treated appropriately [24]. The average outpatients cost in tertiary hospitals was 3.3–5.3 times those in primary health centres, bringing heavy economic burden for hypertensive patients [25]. Actually, hypertension can be managed well by cost-effective primary care interventions instead of being treated in tertiary hospitals [26]. Treating hypertensive patients in tertiary hospitals not only wastes the limited health resources, it also increases the economic burden of hypertensive patients.

Hypertensive patients who are using outpatient services in tertiary hospitals have not been characterised systematically, and healthcare providers and policymakers have insufficient knowledge regarding those populations. Catering to hypertensive patients in the outpatient unit of tertiary hospitals can possibly lead to poor outpatient utilisation management. Previous studies have neither revealed the characteristics of outpatient service utilisation for hypertension in tertiary hospitals nor used longitudinal data to describe the longitudinal characteristics of the trajectories of outpatient service utilisation [27,28,29,30,31]. A few studies on outpatient utilisation patterns used outpatient visit frequency to explore the relationship between pattern and prevalence or the control of hypertension [32,33]. However, these studies did not identify the differences among various trajectories according to outpatient utilisation over time.

In contrast to the traditional method for clustering subject for cross-sectional data, latent class growth analysis (LCGA) was used to identify the latent group with the same characteristics over time [34,35]. LCGA has been widely used in mental health and health service research [36,37,38,39]. However, the trajectories or patterns of the hypertensive-related medical cost of medical service utilisation over time have not been studied yet. Stratifying hypertensive patients into different groups according to outpatient utilisation patterns and comprehensively understanding outpatient service utilisation by hypertensive patients in tertiary hospitals in China greatly help the future development of management programmes and health-promoting strategies.

The prevalence and economic burden of hypertension in China are growing; thus, identifying hypertensive patients with different trajectories in outpatient service utilisation is essential to the enhancement of personalised health management strategies for relieving the economic burden of patients and reducing the waste of health resources. Therefore, the trajectory of outpatient utilisation over time and the association between trajectories and patient characteristics must be explored. The current study has the following main objectives: (1) to characterise hypertensive patients who are using outpatient services in tertiary hospitals; (2) to identify the k number of distinct groups based on the trajectories of outpatient service utilisation using LCGA on a 12-month period; and (3) to investigate the determinants of the trajectory groups of outpatient service utilisation.

## 2. Method

### 2.1. Study Design and Population

Research data were extracted from the Yichang Health Management Centre, where the hospital information system, population information database, and public health information system are interconnected. A unique identification (ID) was given to each patient for recognition and identification in different information systems.

Patients who met the following criteria were selected: (1) diagnosed with hypertension before 1 January 2016 in the criterion of systolic blood pressure (BP) ≥ 140 mm Hg and/or diastolic BP ≥ 90 mm Hg, (2) are subjects of the Hypertension Management Programme, (3) had at least one outpatient encounter with a diagnosis of hypertension-related disease in hospital in 2016, and (4) still living on 31 December 2016.

Hypertensive patients were recognised through the name list from the Hypertension Management Programme. All hypertensive patients’ hypertension-related medical service utilisation records, including outpatient and inpatient service utilisation, were collected from the hospital information system through their ID. In the present study, hypertension-related diseases refer to the diagnosis with ICD-10 codes: I10, I11, I12, I13, and I15 [40,41].

Medical expenditures, including outpatient and inpatient expenditures, were also collected from the hospital information system. Outpatient expenditures refer to the cost generated in outpatient services, including medical services, outpatient examination, and outpatient medicine expenditures, in all hospitals. Inpatient expenditures refer to the cost generated in inpatient services, including expenditures in hospital stay, treatment, examination, laboratory tests, operation, nursing, and medication, in all the hospitals. The costs of self-purchased medicine were not collected in this study due to the lack of information. Since all the patients in this study had participated in social medical insurance and had a similar outpatient reimbursement policy in tertiary hospitals, the factor of medical insurance will not be examined in this study.

This study was approved by the ethics committee of Tongji Medical College, Huazhong University of Science and Technology (IORG No: IOGR0003571). All information and data used in this study were de-identified.

### 2.2. Statistical Analysis

The clusters of individuals over time were identified through latent class growth analysis (LCGA) [42,43]. Different trajectory groups were generated based on the principle that changes in identified clusters should ensure homogeneity within and heterogeneity between clusters for the creation of distinct subgroups [44,45]. Each patient’s monthly hypertension-related outpatient service utilisations were counted. Monthly utilisation frequency is a discrete variable—that is, a variable with countable data and excess number of zeros; hence, a zero-inflated passion model was used in the simulation of the trajectory of outpatient service utilisation [46]. Bayesian information criterion (BIC) and Akaike information criterion (AIC) were calculated for the evaluation of the number of distinct trajectories and selection of the best fit model [47].

Demographic characteristics and medical utilisation were summarised as means with standard deviation for continuous variables and as counts and percentages for categorical variables in different trajectory groups according to the results of LCGA. The chi-square test was used to test the difference of the percentages of categorical variables (sex, marital status, place of residence, education, follow-up, and inpatient service utilisation) among the four identified trajectory groups. One-way ANOVA was used to test the differences of continuous variables (age, outpatient visit times, outpatient cost, and medical cost).

Determinants of the identified trajectories were examined by multinomial logistic regression. The independent effects of age, gender, marital status, place of residence, education level, and medical service utilisation-related variables on the latent trajectories of outpatient service utilisation were examined in the logistic regression model.

All statistical analyses were conducted using Stata 14.0 (StataCorp LP, College Station, TX, USA), and LCGA was conducted through the Proc Traj procedure (https://www.andrew.cmu.edu/user/bjones/). *p* <0.05 was considered significant. All costs were estimated in dollars (US$1 = 6.64 RMB in 2016).

## 3. Results

### 3.1. Basic Characteristics of Hypertensive Patients

The summary of the demographic characteristics and variables related to medical service utilisation are presented in Table 1. The average age of hypertensive patients involved in this study was 64.77 years. Among these patients, 53.7% were male and 64.75% were married. The majority of patients (75.1%) lived in urban areas, and almost half of them (45.85%) received no more than primary education. Only 206 (2.10%) of them had utilised follow-up services in primary healthcare centres. In terms of patient medical service utilisation, the average number of outpatient visits was 5.59 times for all patients, and the median number of service utilisation was 4. A total of 3027 (30.8%) patients had inpatient service utilisation in 2016. The average annual outpatient cost per patient, outpatient cost per visit, and median annual outpatient cost were $227.09, $40.62, and $166.55, respectively. Besides, the average annual medical cost per patient was $985.94, and the median medical cost was $265.38.

### 3.2. Trajectories of Outpatient Service Utilisation among Hypertensive Patients

**Figure 1:**
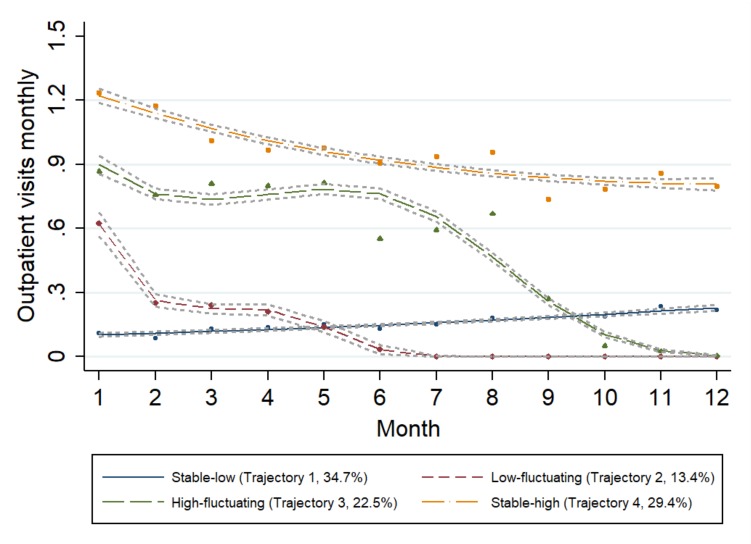
Trajectories of outpatient service utilisation over time (*n* = 9822).

Table 2 shows the model fitting information, including the polynomial order of coefficients, AIC, BIC, and log Bayes factors of the outpatient service trajectories of different classes. As shown by the model fit information, the four clusters (k = 4) would provide the best fit model to describe the heterogeneity of the patterns of outpatient service utilisation. In Figure 1, the four trajectory curves showed the heterogeneity of the patterns of outpatient service utilisation among the four identified groups. The stable-low group (Trajectory 1, *N* = 3330; 34.7% of the sample) was the largest and had stable-low outpatient service utilisation, in which the number of outpatient visits increased slowly over time. The low-fluctuating group (Trajectory 2, *N* = 1519; 13.4%) was characterised by the initial sharp decreasing trend and then the absence of outpatient service utilisation after the middle of the year. The high-fluctuating group (Trajectory 3, *N* = 2279; 22.5%) indicated high outpatient service utilisation in the first half of the year but showed a sharp decrease in the middle of the year. The stable-high group (Trajectory 4, *N* = 2694; 29.4%) maintained the highest outpatient service utilisation than the other trajectories, with a slight decrease over time.

### 3.3. Differences of the Demographic Characteristics and Medical Service Utilisation-Related Variables Among Different Trajectory Groups

The demographic characteristics and variables related to the medical service utilisation of each trajectory group were examined, and the results are presented in Table 1. Demographic characteristics such as the age, gender, marital status, living place, and the education of the four groups were remarkably different. The hypertensive patients in Group 4 had more senior patients (age≥65, 69.6%) than those in Groups 1 (46.9%, χ^2^ = 130.98, *p* < 0.001), 2 (40.7%, χ^2^ = 175.54, *p* < 0.001), and 3 (56.5%, χ^2^ = 11.07, *p* = 0.001). Group 4 also had more males (59.3%) compared with Groups 1 (51.7%, χ^2^ = 36.37, *p* < 0.001), 2 (52.0%, χ^2^ = 21.13, *p* < 0.001), and 3 (51.3%, χ^2^ = 31.85, *p* < 0.001). With regard to marital status, the patients in Group 4 had higher marriage rates (79.4%) than those in Groups 1 (73.4%, χ^2^ = 21.13, *p* < 0.001), 2 (71.7%, χ^2^ = 31.87, *p* < 0.001), and 3 (57.5%, χ^2^ = 276.98, *p* < 0.001). Group 4 had more patients from urban areas (85.2%) than Groups 1 (75.2%, χ^2^ = 92.56, *p* < 0.001), 2 (70.5%, χ^2^ = 131.14, *p* < 0.001), and 3 (66.2%, χ^2^ = 249.35, *p* < 0.001). Regarding medical service utilisation, no significant difference among groups was observed in terms of inpatient cost (*p* = 0.079). The rates of follow-up (χ^2^ = 54.437, *p* < 0.001) and inpatient service utilisation (χ^2^ = 66.02, p < 0.001) were significantly different among the four trajectory groups. Group 3 had the lowest rate of inpatient service utilisation. The differences of the mean of outpatient visit times (F = 12,508.8, *p* < 0.001), outpatient cost (F = 2373.74, *p* < 0.001), and medical cost (F = 15.98, *p* < 0.001) among the groups were also statistically significant. Patients in Trajectory 4 had the highest outpatient visit time, outpatient cost, and medical cost. The patients in Trajectory 2 had the lowest average total outpatient cost. The median outpatient costs of patients in each group were as follows: $53.01 in Group 1, $40.46 in Group 2, $227.85 in Group 3, and $385.30 in Group 4. Patients in Trajectory 2 also had the lowest medical costs, and the median medical cost in each group were as follows: $87.87 in Group 1, $66.05 in Group 2, $245.77 in Group 3, and $463.70 in Group 4.

### 3.4. Determinants of Outpatient Service Utilisation Trajectories

The results of multinomial logistic regression regarding the determinants of trajectory groups are presented in Table 3. As the largest group, Trajectory 1 was specified as the reference category. As shown in Table 3, hypertensive patients who were married had a lower probability of being in Trajectory 3 (relative to Trajectory 1). Patients who live in urban areas had a lower probability of being in Trajectory 2 (relative to Trajectory 1). Patients who had primary and junior high school education had a low probability of being in Trajectory 3 (relative to Trajectory 1). However, those with primary school education had a high probability of being in Trajectory 4 (relative to Trajectory 1). As for medical utilisation, hypertensive patients with high outpatient service utilisation had a low probability of being in Trajectory 2 but a high probability of being in Trajectories 3 or 4. The probability of patients who had inpatient service being in Trajectories 2 and 3 decreased by 17.7% (OR = 0.833, *p* = 0.018) and 32.1% (OR = 0.679, *p* = 0.003), respectively (relative to Trajectory 1). Besides, patients with high outpatient cost had a low probability of being in Trajectories 2 or 3.

## 4. Discussion

To the best of our knowledge, this study is the first to use LCGA to identify the trajectory of outpatient service utilisation of hypertensive patients. Compared with previous cross-sectional studies, this study described the demographic and longitudinal utilisation characteristics of hypertensive patients who are using outpatient services in tertiary hospitals. Four distinct trajectory groups of outpatient service utilisation were identified. These groups were stable-low (Trajectory 1, 34.7%), low-fluctuating (Trajectory 2, 13.4%), high-fluctuating (Trajectory 3, 22.5%), and stable-high (Trajectory 4, 29.4%). The determinants of outpatient utilisation trajectories contributed to the current research on outpatient utilisation patterns. These results may shed light on the proper utilisation of outpatient service in tertiary hospitals and the development of hypertension management.

In line with existing studies, the majority of patients visiting outpatient units in tertiary hospitals were middle-aged or elderly patients and were well-educated [28,31,48,49]. However, patients using outpatient services in primary healthcare institutions were relatively younger than those in visiting tertiary hospitals, because young patients pay less attention to their hypertension management [49]. Obviously, the education level is strongly associated with patients’ awareness of hypertension [49]. Chinese patients with better hypertension awareness prefer large hospitals with better medical quality [29,30]. Therefore, well-educated patients were more likely to use outpatient services in tertiary hospitals instead of primary healthcare institutions due to their preference for high-quality medical service and better financial status [50]. Actually, one-third of hypertensive patients visiting outpatient units in tertiary hospitals have reasonable blood pressure control and thus waste limited medical resources [29]. The follow-up rate of patients in tertiary hospitals is far lower than the national average (56.3%) [51], indicating that hypertension management for those patients needs to be further strengthened. Strengthening the quality of primary healthcare is essential for channelling patients to primary healthcare institutions for outpatient service.

Almost one-third of hypertensive patients in this study were grouped in the stable-low trajectory (34.7%), and nearly one-third of hypertensive patients were grouped in the stable-high trajectory (29.4%). Thus, stable-group patients are more likely to use tertiary hospital outpatient services regularly as their follow-up services. Although patients use medical services in tertiary hospitals for better service, availing these services for hypertension wastes limited medical resources [29]. Conversely, patients in the stable-high trajectory had about a 6 times higher rate of inpatient service utilisation than those in the stable-low trajectory. More than 60% of patients in Trajectory 4 were over 65 years, which may help to explain their greater outpatient utilisation [52]. In terms of outpatient service utilisation, Trajectories 1 and 4 were relatively stable, whereas Trajectories 2 and 3 declined over time. These latter trajectories had high rates of outpatient service utilisation in a period and then decreased rapidly. Therefore, fluctuating-group patients are more like to use outpatient services in tertiary hospitals in emergency and severe conditions. Identifying the trajectory of outpatient service utilisation over time contributes to the intervention and management of patients. Trajectory-specific strategies for the more effective management of hypertension should be highlighted. For patients in Trajectories 1 and 4, the referral mechanism should be improved to manage patients to use follow-up and regular outpatient service in primary healthcare centres, whereas for patients in Trajectories 2 and 3, better health management is needed to control their hypertension.

The demographic characteristics of patients in different trajectories are evident. Consistent with previous studies, the age, marital status, and place of residence were found to be the determinants of outpatient utilisation trajectory [5,53,54,55,56]. Well-educated and married patients are associated with regular outpatient utilisation. These factors may contribute to improving hypertension adherence through the opportunity to self-control and provide material support [55]. However, a higher proportion of rural patients found in Trajectories 2 and 3 had fluctuating outpatient utilisation over time. These patients were more likely to use outpatient services only in severe conditions. According to previous empirical evidence in China, the prevalence of hypertension among adults in rural areas was comparable to that of adults in urban areas, yet the hypertension treatment of adults in rural areas was considerably lower than that of adults in urban areas [55,56]. On the one hand, rural patients cannot use outpatient services in tertiary hospitals as regularly as urban patients because of the distance; on the other hand, a lack of knowledge or awareness of hypertension may lead to the less outpatient utilisation of rural patients [55,56]. Although measures have been taken to narrow the gap between urban and rural areas [55], inequality is still found between hypertension patients in urban and rural areas. Improving health education and strengthening primary care for rural patients and the equalisation of primary care services for hypertension patients between rural and urban areas are essential for better hypertension management.

Medical service utilisation is the leading cause of economic burden for hypertensive patients [14,57,58,59]. In China, patients in tertiary hospitals had significantly higher expenses than patients in primary healthcare institutions [25,60]. However, our results showed that outpatient expenditure and total medical expenditure were significantly different among different trajectory groups of patients. Elderly and well-educated patients have high expenditures because of more medical utilisation [59,61]. The same finding was observed in our study. Trajectories 1 and 3 had similar medical expenditures, but the expenses composition is not similar because of different medical utilisation patterns. Patients in Trajectory Group 3 have higher outpatient service utilisation and lower inpatient service utilisation than the patients in Group 1. The substitution of outpatient cost for inpatient cost had been proved in many studies [27,62]. Preventative healthcare service and hypertension management should be strengthened for patients with high inpatient service utilisation, such as those in Trajectory Group 1, to shift the utilisation from inpatient to outpatient. Furthermore, the unnecessary utilisation of outpatient services in tertiary hospitals needs to be relayed to primary healthcare institutions to reduce economic burden.

These results indicated that hypertensive patients in different trajectory groups of outpatient utilisation have various characteristics and medical expenditures. Hypertensive patients with comorbidity or multimorbidity spend more than their counterparts [58,61,63]. Patients in Trajectory Group 4 had the highest medical expenditure, which was possibly because of the complexity of the hypertension condition. Medical treatment is necessary for patients with uncontrolled hypertension, especially for the elderly and those with comorbidity and multimorbidity [61,64,65]. Uncontrolled hypertension leads to complications such as stroke, which increases medical utilisation and total expenditure [59,66]. Multimorbidity has a high utilisation of inpatient care and accounts for healthcare inefficiency [67,68]. The high utilisation and expenditure of hypertensive patients such as those in the high-stable group need additional attention. Targeting measures in the hypertension management programme should be taken for these high-risk group patients to achieve a higher adherence to medical treatment and effectively reduce medical cost.

This study has several limitations. Firstly, this study was conducted to explore the trajectory and determinants of outpatient service utilisation of hypertensive patients over time. However, health status and hypertension complications were not included. Patients with different disease conditions may have different needs of medical service utilisation and health outcomes. Secondly, the medical insurance information of patients was not collected in the Yichang Health Management Centre. Different medical insurance may influence patients’ decision-making on medical service utilisation [69,70]. Thirdly, medication expenditures in drug stores constitute a considerable proportion of the total medical expenditure of hypertensive patients [71,72]. Self-medication is a crucial component of the service utilisation of these patients. However, this study did not collect the self-medication expenditures of patients, and thus biases may have occurred in the examination of the relationship between utilisation patterns and medical expenditures.

## 5. Conclusions

In conclusion, hypertensive patients visiting outpatient units in tertiary hospitals were middle-aged, elderly, and well-educated, and they received poor follow-up services. However, outpatient utilisation patterns, especially longitudinal characteristics, varied among those patients. The four trajectories of longitudinal outpatient utilisation of hypertensive patients were identified: stable-low, low-fluctuating, stable-high, and high-fluctuating. Patients in the stable group preferred to visit outpatient units in tertiary hospitals as their follow-up service, whereas those in the fluctuating group preferred to visit outpatient units in tertiary hospitals in emergency and severe conditions. Visiting outpatient patterns between patients in urban and rural areas and the utilisation of outpatient and inpatient services were different among trajectory groups. Trajectory group-based measurements are needed for hypertension management and economic burden reduction because of different utilisation patterns. Patients with high inpatient utilisation need to be managed intensively through a hypertension management programme for the reduction of inpatient utilisation and encouraging the patients to visit outpatient units in primary healthcare institutions. Further studies should take patients’ complications and comorbid conditions into consideration to accurately describe the relationship between patients’ outpatient utilisation trajectory and their health status. Socioeconomic characteristics such as income and medical insurance should also be considered in further studies to help formulate specifically customised intervention measures for people with different socioeconomic characteristics.

## Figures and Tables

**Table 1:** Demographic characteristics by hypertension patient outpatient service utilisation trajectories.

Variables	Overall (*N* = 9822)	Trajectory 1 (*N* = 3330)	Trajectory 2 (*N* = 1519)	Trajectory 3 (*N* = 2279)	Trajectory 4 (*N* = 2694)	χ2/F	*p*-Value
Age							
<45	778, 7.9%	388, 11.7%	233, 15.3%	79, 3.5%	78, 2.9%	446.24	*p* < 0.001
45–64	3953, 40.2%	1380, 41.4%	669, 44.0%	911, 40.0%	993, 36.9%		
65–80	4109, 41.8%	1289, 38.7%	490, 32.3%	985, 43.2%	1345, 49.9%		
>80	982, 10.0%	273, 8.2%	127, 8.4%	304, 13.3%	278, 10.3%		
Gender							
Male	5278, 53.7%	1720, 51.7%	790, 52.0%	1170, 51.3%	1598, 59.3%	46.67	*p* < 0.001
Female	4544, 46.3%	1610, 48, 3%	729, 48.0%	1109, 48, 7%	1096, 40.7%		
Marital status							
Married	6983, 71.1%	2445, 73.4%	1089, 71.7%	1311, 57.5%	2138, 79.4%	302.85	*p* < 0.001
Others	2839, 28.9%	885, 26.6%	430, 28.3%	968, 42.5%	556, 20.6%		
Place of residence							
Urban	7379, 75.1%	2504, 75.2%	1071, 70.5%	1508, 66.2%	2296, 85.2%	262.27	*p* < 0.001
Rural area	2443, 24.9%	826, 24.8%	448, 29.5%	771, 33.8%	398, 14.8%		
Education							
University	540, 5.5%	206, 6.2%	92, 6.1%	82, 3.6%	160, 5.9%	442.24	*p* < 0.001
Senior high school	2518, 25.6%	862, 25.9%	396, 26.1%	622, 27.3%	638, 23.7%		
Junior high school	2264, 23.1%	793, 23.8%	332, 21.9%	324, 14.2%	815, 30.3%		
Primary school	2264, 23.1%	407, 12.2%	174, 11.5%	171, 7.5%	430, 16.0%		
Others	3318, 33.8%	1062, 31.9%	525, 34.6%	1080, 47.4%	651, 24.2%		
Follow-up	206, 2.1%	103, 3.1%	50, 3.3%	25, 1.1%	28, 1.0%	52.43	*p* < 0.001
Outpatient visit times	5.59 (4.729)	1.88 (1.22)	1.49 (0.79)	6.53 (1.86)	11.70 (3.44)	12508.8	*p* < 0.001
Inpatient service utilisation	3027, 30.8%	1146, 34.4%	444, 29.2%	562, 24.7%	875, 32.5%	66.02	*p* < 0.001
Outpatient cost	227.09 (262.12)	85.64 (163.26)	60.49 (70.00)	244.21 (137.99)	481.39 (305.62)	2373.74	*p* < 0.001
Inpatient cost	758.85 (2467.85)	826.73 (2556.89)	725.84 (2780.33)	657.79 (2646.32)	779.05 (1957.79)	2.265	0.079
Medical cost	985.74 (2497.20)	912.37 (2574.25)	786.34 (2783.53)	902.00 (2663.67)	1260.44 (2017.52)	15.98	*p* < 0.001

The mean value or proportion of each variable of demographic characteristic and medical utilisation were calculated in four outpatient service utilisation trajectories. Proportion was calculated for each categorical variable in the whole sample group and four trajectory groups. Categorical variable (gender, marital status, place of residence, education, follow-up, inpatient service utilisation) were tested for significant differences using Pearson’s chi-square test. One-way ANOVA was used to test the significant difference for continuous variables (age, outpatient visit times, outpatient cost, and medical cost). # Significant results of pairwise comparisons: Age ≥ 65: Group 4 > Group 1 (χ2 = 130.98, *p* < 0.001), Group 4 > Group 2 (χ2 = 175.54, *p* = 0.001); Male: Group 4 > Group 1 (χ2 = 36.37, *p* < 0.001), Group 4 > Group 2 (χ2 = 21.13, *p* < 0.001), Group 4 > Group 3 (χ2 = 31.85, p < 0.001); Married: Group 4 > Group 1 (χ2 = 21.13, p < 0.001), Group 4 > Group 2 (χ2 = 31.87, *p* < 0.001), Group 4 > Group 3 (χ2 = 276.98, *p* < 0.001); Residence of urban: Group 4 > Group 1 (χ2 = 92.56, *p* < 0.001), Group 4 > Group 2 (χ2 = 131.14, *p* < 0.001), Group 4 > Group 3 (χ2 = 249.35, *p* < 0.001).

**Table 2:** Result of model fit statistics.

Number of Class	Polynomial Order of Coefficients	BIC	AIC	Log Bayes Factor
Hypertension patient outpatient service utilisation trajectory				
1	1	−100,803.52	−100,797.61	100,795.61
2	22	−92,401.84	−92,376.67	−92,369.67
3	232	−91,291.47	−91,248.32	−91,236.32
4	1332	−91,036.24	−90,978.7	−90,962.7

BIC = Bayesian Information Criterion; AIC = Akaike Information Criterion.

**Table 3:** Multinomial logistic regression analysis in outpatient service utilisation trajectory groups.

Variables	Trajectory 2			Trajectory 3			Trajectory 4		
	β	OR (95%CI)	*p*-Value	β	OR(95%CI)	*p*-Value	β	OR(95%CI)	*p*-Value
Age									
≤45	0.071	1.074 (0.812–1.420)	0.616	−0.655	0.519 (0.295–0.915)	0.023	0.152	1.165 (0.539–2.517)	0.698
45–65	−0.055	0.947 (0.745–1.203)	0.656	−0.245	0.783 (0.541–1.133)	0.194	0.123	1.130 (0.709–1.801)	0.606
65–80	−0.206	0.814 (0.640–1.036)	0.094	−0.459	0.632 (0.439–0.909)	0.013	−0.240	0.786 (0.498–1.241)	0.302
Gender	0.039	1.039 (0.918–1.177)	0.544	−0.095	0.910 (0.729–1.135)	0.403	0.140	1.151 (0.869–1.523)	0.326
Marital (married)	0.018	1.018 (0.859–1.207)	0.835	−0.327	0.721 (0.527–0.987)	0.041	0.221	1.247 (0.836–1.859)	0.279
Place of residence (urban area)	−0.189	0.828 (0.710–0.965)	0.016	−0.244	0.784 (0.584–1.051)	0.103	0.098	1.103 (0.751–1.619)	0.617
Education									
University	−0.087	0.917 (0.686–1.227)	0.560	−0.318	0.727 (0.410–1.291)	0.277	0.329	1.390 (0.680–2.838)	0.367
Senior high school	0.012	1.012 (0.836–1.225)	0.905	0.105	1.111 (0.788–1.566)	0.549	0.043	1.044 (0.674–1.617)	0.847
Junior high school	−0.121	0.886 (0.733–1.070)	0.209	−0.474	0.622 (0.437–0.887)	0.009	0.398	1.489 (0.955–2.321)	0.079
Primary school	−0.025	0.976 (0.777–1.226)	0.832	−0.526	0.591 (0.389–0.896)	0.013	0.567	1.763 (1.044–2.976)	0.034
Follow-up	0.060	1.062 (0.745–1.513)	0.739	−0.034	0.967 (0.450–2.076)	0.931	0.259	1.295 (0.440–3.810)	0.639
Outpatient visit times,	−0.335	0.715 (0.663–0.772)	<0.001	1.652	5.219 (4.748–5.737)	<0.001	2.460	11.700 (10.524–13.007)	<0.001
Inpatient service utilisation	−0.182	0.833 (0.717–0.969)	0.018	-0.387	0.679 (0.525–0.879)	0.003	0.049	1.051 (0.748–1.476)	0.776
Outpatient cost	−0.888	0.412 (0.179–0.944)	0.036	-1.391	0.249 (0.123–0.502)	<0.001	0.313	1.367 (0.733–2.549)	0.325
Total cost	0.009	1.009 (0.983–1.036)	0.492	0.021	1.021 (0.982–1.062)	<0.001	0.026	1.026 (0.967–1.090)	0.393

The risk profile for each trajectory group compared to trajectory Group 1 (stable-low and increasing slowly). Predictors in bold are those that were statistically significant. OR, odds ratio; CI, confidence interval. Reference: (1) marital status: other; (2) place of residence: other; (3) education: other; (4) inpatient service utilisation: without inpatient service utilisation; Outpatient cost use per thousand dollars.
